# The Benefits of Crowdsourcing to Seed and Align an Algorithm in an mHealth Intervention for African American and Hispanic Adults: Survey Study

**DOI:** 10.2196/30216

**Published:** 2022-06-21

**Authors:** Neil Jay Sehgal, Shuo Huang, Neil Mason Johnson, John Dickerson, Devlon Jackson, Cynthia Baur

**Affiliations:** 1 Department of Health Policy and Management School of Public Health University of Maryland College Park, MD United States; 2 Horowitz Center for Health Literacy School of Public Health University of Maryland College Park, MD United States; 3 Department of Computer Science University of Maryland College Park, MD United States; 4 Department of Behavioral and Community Health School of Public Health University of Maryland College Park, MD United States

**Keywords:** crowdsourcing, health information, health promotion, prevention, public health informatics, African American, Black, Latino, and Hispanic populations, recommender system, RecSys, machine learning, Mechanical Turk, MTurk, mobile phone

## Abstract

**Background:**

The lack of publicly available and culturally relevant data sets on African American and bilingual/Spanish-speaking Hispanic adults’ disease prevention and health promotion priorities presents a major challenge for researchers and developers who want to create and test personalized tools built on and aligned with those priorities. Personalization depends on prediction and performance data. A recommender system (RecSys) could predict the most culturally and personally relevant preventative health information and serve it to African American and Hispanic users via a novel smartphone app. However, early in a user’s experience, a RecSys can face the “cold start problem” of serving untailored and irrelevant content before it learns user preferences. For underserved African American and Hispanic populations, who are consistently being served health content targeted toward the White majority, the cold start problem can become an example of algorithmic bias. To avoid this, a RecSys needs population-appropriate seed data aligned with the app’s purposes. Crowdsourcing provides a means to generate population-appropriate seed data.

**Objective:**

Our objective was to identify and test a method to address the lack of culturally specific preventative personal health data and sidestep the type of algorithmic bias inherent in a RecSys not trained in the population of focus. We did this by collecting a large amount of data quickly and at low cost from members of the population of focus, thereby generating a novel data set based on prevention-focused, population-relevant health goals. We seeded our RecSys with data collected anonymously from self-identified Hispanic and self-identified non-Hispanic African American/Black adult respondents, using Amazon Mechanical Turk (MTurk).

**Methods:**

MTurk provided the crowdsourcing platform for a web-based survey in which respondents completed a personal profile and a health information–seeking assessment, and provided data on family health history and personal health history. Respondents then selected their top 3 health goals related to preventable health conditions, and for each goal, reviewed and rated the top 3 information returns by importance, personal utility, whether the item should be added to their personal health library, and their satisfaction with the quality of the information returned. This paper reports the article ratings because our intent was to assess the benefits of crowdsourcing to seed a RecSys. The analysis of the data from health goals will be reported in future papers.

**Results:**

The MTurk crowdsourcing approach generated 985 valid responses from 485 (49%) self-identified Hispanic and 500 (51%) self-identified non-Hispanic African American adults over the course of only 64 days at a cost of US $6.74 per respondent. Respondents rated 92 unique articles to inform the RecSys.

**Conclusions:**

Researchers have options such as MTurk as a quick, low-cost means to avoid the cold start problem for algorithms and to sidestep bias and low relevance for an intended population of app users. Seeding a RecSys with responses from people like the intended users allows for the development of a digital health tool that can recommend information to users based on similar demography, health goals, and health history. This approach minimizes the potential, initial gaps in algorithm performance; allows for quicker algorithm refinement in use; and may deliver a better user experience to individuals seeking preventative health information to improve health and achieve health goals.

## Introduction

### Algorithm Personalization

Algorithms are increasingly used to personalize recommendations of items in stored databases. In simple terms, a personalization algorithm is a computer-implemented service that recommends items to a user based on the known characteristics of that user and the historical preferences of other similar users. The process of training a personalization algorithm is a type of machine learning. The resulting personalization tool is in effect a recommender system (RecSys)—a collaborative information filtering system that attempts to predict a user’s preferences for an item based on the previously recorded, similar preferences of other users. Collaborative filtering underlies many popular implementations of personalization algorithms including Amazon.com’s “people who buy x also buy y” recommendations [1]. In public health, algorithms to offer targeted and personalized health advice based on personal risk profile and patterns of behavior are as yet an unrealized opportunity [2].

To avoid problems of early poor performance in a new RecSys, algorithms are frequently trained using publicly available data prior to being applied. However, algorithms may reproduce racial, ethnic, and gender disparities because of the data used to train them [3,4]. Racial bias has been detected in commercial algorithms used to guide health decisions among providers [5], as well as in algorithms for hiring [6], natural language processing [7], and sentencing and parole guidelines [8,9]. Algorithms trained on large population-level data sets may underperform when personalizing recommendations for diverse populations [3]. When recommending preventative health information, such underperformance may compound existing inequities in health. The risk of bias inherent in existing publicly available health information data sets is potentially high [3]. Previous qualitative work on barriers to African American and Hispanic adults’ health information seeking has shown that commonly available health information resources can be racially or culturally insensitive or may be written implicitly for the dominant culture and not be culturally relevant for the intended population of users [10,11]. A RecSys trained on a data set with very few African American or Hispanic participants may cause these culturally inappropriate resources to be promoted rather than demoted by that RecSys [3].

The lack of publicly available data sets for Black and bilingual/Spanish-speaking Hispanic users of health websites presents a major challenge to researchers who want to develop personalized tools for the health behavior intervention space. Our searches (conducted repeatedly on all dates between November 2020 and November 2021) for “training data,” “training data set,” “seed data,” “collaborative filtering,” or “recsys,” paired with “black,” “african american,” “latino,” “hispanic,” or “race” returned no relevant results or data sets for health information seeking in PubMed and Google Scholar. The time and cost required to collect sufficient new population-specific data to seed an algorithm are additional barriers, especially when the need is for 2 different population groups using 2 different languages, such as English and Spanish.

A potential common means of controlling algorithmic bias is “masking” the algorithm to race or gender in order to avoid capturing or exacerbating any social or structural inequity reflected in the training data. This process of excluding race or gender might solve the algorithmic bias problem in other domains where an algorithm is employed to assist in a decision-making process orthogonal to the demographic characteristic excluded. However, personalization in mobile health (mHealth) depends specifically on race- or gender-based predictions, as race, ethnicity, and gender are key social determinants of health [12]. “Fair” algorithms focused on health must account for the diversity of the groups of people the algorithm’s performance may affect [5], and as such, algorithmic fairness in health requires a solution other than masking. Instead of using potentially biased training data or ignoring the impact of race and ethnicity on health, researchers and practitioners need to be able to generate, share, and use robust seed data gathered from people similar to the intended users who will be affected by the algorithm’s outputs.

### Background

The RecSys seeding discussed in this paper is part of a 4-year smartphone health app research study funded by the National Library of Medicine (Grant 5R01LM013039-02), titled “HealthyMe/MiSalud Smartphone Application: Identifying Mechanisms to Engage African Americans and Hispanics in Personal Health Libraries.” A University of Maryland Center for Health Literacy research team is developing the RecSys to deliver personalized health content from MyHealthfinder website to English-speaking African Americans and Spanish-speaking Hispanic adults. The MyHealthfinder website is a free, no-copyright consumer health information collection in English and Spanish maintained by the United States Department of Health and Human Services. The team chose the MyHealthfinder website because the website applies health literacy principles and extensive consumer testing rather than limited, mechanistic reading grade formulas [13]. All articles are written in plain language consistent with the Federal Plain Language Guidelines [14] and health literacy criteria in the Centers for Disease Control and Prevention (CDC) Clear Communication Index [15] and cover a wide range of health topics linked to evidence-based recommendations from key federal advisory committees. The MyHealthfinder website allows basic personalization of health articles and prevention recommendations when users enter their age, sex, and pregnancy status. The content is available through an application programming interface.

Our research team planned to use the RecSys as the core of a smartphone app with individualized recommendations, guidance on seeking further information, and capacity for users to build personalized libraries in the app [16]. One of the more frequent applications of data science is to build a RecSys with the principal capacity to predict what a user might do next with a high degree of accuracy and to provide a small set of recommended items that have a high likelihood of attracting the user [17]. Health information providers have lagged behind this trend [18].

Personalization in mHealth depends on prediction and performance data, and algorithms that utilize collaborative filtering either rely on existing data for training or are subject to the cold start problem. The cold start problem happens when insufficient data exist at the launch of a RecSys to ensure high-quality recommendations [19]. Consequently, an inadequately personalized algorithm limits the effectiveness of personalization and the utility of the RecSys itself [19]. Two associated problems with collaborative filtering algorithms are scalability and sparsity, particularly in large data sets [20]. The larger the data set, the more computational power is needed to calculate recommendations and the fewer the items any individual user will rate [20]. Scalability and sparsity also slow the process of algorithm learning; to overcome these challenges, developers often employ an initial seed data set for algorithm training. Seed data are necessary to mitigate the cold start problem. However, using data that are a poor match with the intended user group or that have implicit or explicit biases will undermine the user experience, as well as personalization, and thus the utility of a RecSys [3].

To develop a RecSys to predict the most relevant preventative health information and serve it to African American and Hispanic users, we needed seed data describing the users’ health goals and the associated relevance of articles and topics in the MyHealthfinder website.

### Crowdsourcing

Generating a seed data set is possible with crowdsourcing and the web-based platforms for crowdsourcing tasks used for web-based research [21-23]. Crowdsourcing refers to a set of potential processes through which tasks are proposed by an initiator to solve a problem and are completed by a crowd of individuals rather than a single individual or entity [24]. The components of the crowd operate outside of the initiator’s direct control as represented by traditional, hierarchical, organizational structures [24]. The benefits to the initiator include completion of the tasks and solutions to the problem through the expertise of a crowd that would otherwise be cost- and time-prohibitive under traditional models for organizing labor [24].

Amazon Mechanical Turk (MTurk) has become increasingly popular as a crowdsourcing platform for conducting web-based research involving surveys, as MTurk facilitates access to a large and diverse participant population at a relatively low cost to investigators [21-23]. MTurk functions as a web-based labor market where registered workers complete web-based Human Intelligence Tasks (HITs) to be paid. HITs can include a range of tasks including responding to surveys, manually categorizing complex data, or transcribing data. During registration, all MTurk workers are required to electronically sign a participation agreement confirming that they are at least 18 years of age. Likewise, individual researchers must register as MTurk requesters to post HITs and collect data from consenting workers. MTurk provides a template for the construction of HIT surveys run directly on Amazon’s developer platform [25]. Researchers post HITs on the Amazon marketplace that MTurk workers self-select and can set both inclusion criteria and task completion criteria. Since MTurk workers are preregistered and come from a large pool, using MTurk may help avoid many of the recruitment barriers that slow survey collection.

In aggregating seed data for an mHealth app, MTurk presents a similar challenge to other population-based surveys: while substantially gender balanced, the majority of the US MTurk workers are White compared with the general population [26,27]. However, researchers can account for this by setting inclusion criteria to garner responses from the population of focus, in our case, African American or Hispanic MTurk workers.

## Methods

### Overview

We used the following inclusion criteria to identify MTurk respondents for our study: (1) self-identify as African American/Black or Hispanic/Latino/Latina/Latine; (2) own a smartphone; and (3) are located in the United States. Using MTurk we were able to balance respondents by race or ethnicity. Tasks were completed in a single session. If a participant did not complete the full task, the data were not returned, and there was no cost to the project. Respondents could technically complete the full task by entering invalid data for certain text entry fields. To address this, we excluded from analyses any retained responses where invalid data were entered into text entry fields. The reliance on a single encounter and the monetary incentive for completing the HIT are powerful retention strategies. To characterize respondents, we collected self-reported demographics (race or ethnicity, age, self-identified sex, educational attainment) and 3 health behaviors (BMI, smoking, and alcohol consumption).

Our tasks for each MTurk worker included completing the following: (1) personal health profile; (2) family health history; (3) a series of questions about the experience and frustrations in finding and using health information based on the Health Information National Trends Survey fielded by the National Cancer Institute; (4) choosing 3 goals from a list of 24 derived from the Healthy People 2020 survey, part of the US 10-year health objectives; (5) reading 3 randomly selected, topically relevant articles from the MyHealthfinder web-based database for each of the 3 selected goals; (6) rating each of the 9 articles on two 5-point Likert scales on the importance of the information and feasibility of using the information as well as 1 dichotomous scale on whether or not the respondent would choose to retain the article in a personal library; (7) reading 6 entirely random articles from MyHealthfinder website that may or may not be topical; (8) rating each of those 6 articles using the same 2 Likert and 1 dichotomous scales; (9) searching through the web-based database of the MyHealthfinder website for information relating to each of the 3 goals; and (10) rating each of the information returns, up to 3 from each of the 3 searches, using the same 2 Likert and 1 dichotomous scales. For each MTurk worker who completes the full task (all 10 components), the Amazon marketplace returns an MTurk ID and the data generated.

Among these tasks, article ratings were most important for training an algorithm. In particular, having responses about article relevance was helpful to secure unbiased and population-focused seed data. The outputs of the other HITs are also useful for informing app development but are less directly relevant to seeding a RecSys. Because this is a methodology paper focused on crowdsourcing data for RecSys development, the results of the other outputs are not reported in the next section.

In terms of data collection efficiencies to seed an algorithm, the ability to quickly collect data at a low cost per user is an important consideration. We recorded the time spent on data collection in days and the total cost (including MTurk fees as well as the cost for completed surveys excluded due to invalid data) and calculated the cost per usable respondent.

All analyses were done in Stata/MP software (version 16; StataCorp), SciPy (version 1.6.0; SciPy), and Google Sheets (Google LLC).

### Ethical Considerations

The University of Maryland College Park institutional review board determined this project was exempt from institutional review board review and approval, as no identifiable private information was collected or retained by the research team, and so it did not meet the definition of human subject research.

## Results

Our MTurk crowdsourcing approach produced sufficient data on participant characteristics and expressed the preferences needed to seed the algorithm, assess the cost effectiveness of the data collection method, and address algorithmic implicit bias. These included (1) producing an adequate sample size of populations traditionally with limited data, (2) reducing the data collection period and data collection cost, and (3) collecting specifically the data set required to seed an algorithm and minimize the cold start problem.

### MTurk Benefit 1: Producing an Adequate Sample Size of Populations Traditionally With Limited Data

Our sampling approach produced 2578 respondents who selected and started the survey and a total of 1015 respondents who met the inclusion criteria and completed the full task. We collected and retained data from 1015 respondents out of which 30 respondents (3% of the retained sample) were excluded due to invalid data entered, for a final sample size of 985 (Table 1). A total of 500 (51%) respondents identified as non-Hispanic Black or African American and 485 (49%) identified as Hispanic/Latino/Latina/Latine. There was an almost even split between self-identified female and male respondents, and 3 respondents (less than 1%) of the sample did not identify with the binary gender designations. Respondents tended to be younger, with a mean age of 32 (SD 9) years, and 545 (55%) of the sample were between the ages of 18 and 30 years. Potentially reflective of the younger age and online recruitment of respondents, 830 (83%) respondents reported having at least some college education, of those 239 (24%) had completed college or a graduate degree.

**Table 1:** Self-reported participant demographics.

Characteristics			All participants (N=985)
**Race/Ethnicity^a^, n (%)**			
	Non-Hispanic Black	500 (50.76)	
	Hispanic/Latino/Latina/Latine	485 (49.24)	
**Age (years), mean (SD)**			32.15 (8.75)
	18-30, n (%)	545 (54.50)	
	31-40, n (%)	305 (30.50)	
	41-50, n (%)	105 (10.50)	
	51-60, n (%)	37 (3.70)	
	61-70, n (%)	8 (0.80)	
**Sex, n (%)**			
	Female	494 (49.45)	
	Male	502 (50.25)	
	Other	3 (0.30)	
**BMI^b^, mean (SD)**			26.64 (12.64)
	Underweight, n (%)	94 (9.64)	
	Normal, n (%)	363 (37.23)	
	Overweight, n (%)	267 (27.38)	
	Obese, n (%)	251 (25.76)	
**Drink 2** **×** **/week, mean (SD)**			294 (39.30)
**Currently smoker, mean (SD)**			194 (19.62)
**Educational level, n (%)**			
	High school or lower	165 (16.58)	
	Some college	591 (59.40)	
	College degree	115 (11.56)	
	Graduate degree	124 (12.46)	

^a^Non-Hispanic Black and Latino/Latina/Latine are derived from self-reported race and Hispanic ethnicity items.

^b^BMI was calculated using height, weight, and sex, and using BMI English system on the Center for Disease Control and Prevention website. The ranges were devised by the World Health Organization.

### MTurk Benefit 2: Reducing the Data Collection Period and the Data Collection Cost

It took 64 days to collect data for the training set. The total cost including MTurk fees and the cost for 30 unusable respondents was US $6635.20 or US $6.74 per usable respondent. An alternative data collection method resulting in 985 unique respondents would have likely taken considerably longer and incurred substantially greater expenses. Alternatively, seeding our algorithm with data from fewer unique respondents would not have adequately minimized the cold start problem.

### MTurk Benefit 3: Collecting Specifically the Data Set Required to Seed the Algorithm and Minimize the Risk of the Cold Start Problem

Respondents rated a total of 92 unique articles. A selection of the top 5 articles that Black and Hispanic respondents rated by importance and by feasibility of using the information is presented in Table 2.

**Table 2:** Comparison of Black and Hispanic participants: the top 5 rated articles on the MyHealthfinder website.

Rating^a^			Article^b^ name	
			Black participants	Hispanic participants
**Importance**				
	1st	Reduce Your Risk of Stroke		Reduce Your Risk of Stroke
	2nd	Prevent Infections When You Get Medical Care		Get Your Blood Pressure Checked
	3rd	Manage Stress		Talk with Your Doctor about Taking Aspirin to Prevent Disease
	4th	Quit Smoking		Manage Stress
	5th	Get Screened		Take Care of Your Teeth and Gums
**Feasibility**				
	1st	Reduce Your Risk of Stroke		Reduce Your Risk of Stroke
	2nd	Learn First Aid		Manage Stress
	3rd	Get Screened		Talk with Your Doctor about Taking Aspirin to Prevent Disease
	4th	Manage Stress		Quit Smoking
	5th	Prevent Infections When You Get Medical Care		Get Your Blood Pressure Checked

^a^Respondents rated importance and feasibility for each article on a 5-point Likert scale. Importance and feasibility are measured on a range of 1 to 5, derived from the Health Information National Trends Survey. A total of 92 unique articles were rated. We have displayed the top 5 articles by importance and feasibility for each demographic group.

^b^Articles were pulled from the MyHealthfinder website and were read and rated by the respondents.

## Discussion

### Principal Findings

Previous studies have shown that crowdsourcing is an effective means of gathering data from a large number of human participants quickly and at a low cost [21-23]. Our results show that crowdsourcing through a technology such as Amazon MTurk can leverage a large, low-cost sampling method to generate seed data for a RecSys and sidestep the cold start problem and the potential algorithmic racial bias inherent in using general population seed data [3]. Unlike traditional survey methods that are reliant on a response rate, the MTurk approach ensures that required cohort sizes are met as HITs remain open until prespecified participant thresholds are met, and the researcher receives data only on respondents who complete all data collection tasks.

Our approach also allows for the development of a digital health tool to recommend more relevant information to users based on similar demography and health history. This is particularly important for public health purposes, where both algorithmic bias and the common tactic of masking algorithms to demographic data might limit the utility of a prevention-focused mHealth tool [3-5]. Through crowdsourcing we were able to efficiently and affordably recruit a large sample of African American and Hispanic participants—our population of focus—to share their health goals and for each goal, rate article returns from a federally supported database of public health information. In addition, the results of the HITs that are not reported in this methodology paper also informed app design and developments beyond the RecSys.

Along with far greater flexibility in item content and greater timeliness, the cost per usable response was an order of magnitude below the cost per complete response (US $40 to US $102) compared with similarly detailed health questionnaires such as the Behavioral Risk Factor Surveillance System (BRFSS) survey [28]. Our data collection period of around 2 months is far more condensed than the BRFSS’s year-round data collection. To our knowledge, we have collected the first such publicly available seed data set for health information seeking for non-Hispanic African American and Hispanic populations.

### Limitations

The principal limitation of this study is that despite a large sample size and despite limiting data collection to African American and Hispanic respondents, MTurk participants are potentially demographically dissimilar in some ways to our app user population. On average, MTurk workers are younger and more educated than the general population and are likely more technologically literate as demonstrated by their participation as workers in a web-based marketplace. However, the majority of our respondents did not have a 4-year or graduate degree. A total of 756 (76%) respondents had only some college education or less, which was similar to our intended app user group. Studying the deployment of the HealthyMe/MiSalud RecSys trained on these seed data will allow us to quantify to what extent these demographic differences limited the applicability of preventative health information provided by the personal health app.

In our deployment, it is not imperative, however, that the seed data perfectly match the intended app user population, since the RecSys continues to “learn” iteratively as app users review and rate articles, further refining the recommendations that the system makes. Importantly, in this way the limitation inherent in crowdsourcing with MTurk does not pose a significant impact on the development of a RecSys, and the benefits of demographically similar (though not identical) seed data in overcoming the cold start problem, scalability, and sparsity likely exceed the limitations of training the RecSys with MTurk data. Future evaluations and field tests of our RecSys will enable us to quantify the utility of a crowdsourced population-specific seeded RecSys versus a generically seeded RecSys or an unseeded RecSys in returning user-rated relevancy of personalized health content and improving user health information-seeking behaviors in these populations.

### Conclusion

Researchers have crowdsourcing options such as Amazon MTurk, for quick, low-cost means to avoid the cold start problem for algorithms and sidestep bias and low relevance for an intended population of app users. Seeding a RecSys with more population-relevant responses allows for the development of a digital health tool that can recommend more relevant information to users based on similar demography, health goals, and health history. If made publicly available, the generation of such seed data sets can also enable other researchers and developers to more rapidly develop additional population-specific solutions for health and health literacy. In the long term, this approach may minimize potential initial gaps in algorithm performance, allow quicker algorithm refinement, and deliver a better user experience.

## Abbreviations

BRFSS: Behavioral Risk Factor Surveillance System
CDC: Centers for Disease Control and Prevention
HIT: Human Intelligence Task
mHealth: mobile health
MTurk: Amazon Mechanical Turk
RecSys: recommender system
